# Two Novel Variants in Genes of Arrhythmogenic Right Ventricular Cardiomyopathy – a Case Report

**DOI:** 10.15388/Amed.2020.28.1.1

**Published:** 2021-01-18

**Authors:** Dovilė Gabartaitė, Dovilė Jančauskaitė, Violeta Mikštienė, Eglė Preikšaitienė, Rimvydas Norvilas, Nomeda Valevičienė, Germanas Marinskis, Audrius Aidietis, Jūratė Barysienė

**Affiliations:** Faculty of Medicine, Vilnius University, Vilnius, Lithuania Centre of Cardiology and Angiology, Vilnius University Hospital Santaros Klinikos, Vilnius, Lithuania; Faculty of Medicine, Vilnius University, Vilnius, Lithuania Centre of Cardiology and Angiology, Vilnius University Hospital Santaros Klinikos, Vilnius, Lithuania; Department of Human and Medical Genetics, Faculty of Medicine, Vilnius University, Vilnius, Lithuania; Department of Human and Medical Genetics, Faculty of Medicine, Vilnius University, Vilnius, Lithuania; Hematology, Oncology and Transfusion Medicine Center, Vilnius University Hospital Santaros Klinikos, Vilnius, Lithuania; Centre of Radiology and Nuclear Medicine, Vilnius University Hospital Santaros Klinikos, Vilnius, Lithuania; Faculty of Medicine, Vilnius University, Vilnius, Lithuania Centre of Cardiology and Angiology, Vilnius University Hospital Santaros Klinikos, Vilnius, Lithuania; Faculty of Medicine, Vilnius University, Vilnius, Lithuania Centre of Cardiology and Angiology, Vilnius University Hospital Santaros Klinikos, Vilnius, Lithuania; Faculty of Medicine, Vilnius University, Vilnius, Lithuania Centre of Cardiology and Angiology, Vilnius University Hospital Santaros Klinikos, Vilnius, Lithuania

**Keywords:** arrhythmogenic right ventricular cardiomyopathy, ventricular arrhythmias, desmosomal mutations

## Abstract

**Summary. Background.:**

Arrhythmogenic right ventricular cardiomyopathy (ARVC) is a heritable cardiomyopathy, characterized by fibrofatty replacement of myocytes in the right ventricular, left ventricular or both ventricles. It is caused by pathogenic variants of genes encoding desmosomal (*JUP*, *DSP*, *PKP2*, *DSG2*, *DSC2) *and non-desmosomal proteins, and is one of the most common causes of sudden cardiac death in young athletes. Therefore, early identification, correct prevention and treatment can prevent adverse outcomes.

**Case report.:**

Our case presents a 65-years-old man with recurrent ventricular tachycardia. The ischemic cause was the first to rule out. Echocardiography revealed right ventricular structural and functional abnormalities. After suspicion of ARVC, magnetic resonance imaging was performed showing reduced right ventricular ejection fraction with local aneurysms, structural changes ir the right and left myocardium. Subsequently performed genetic testing identified a novel ARVC likely pathogenic variant* in DSC2* gene and variant of uncertain significance in *RYR2* gene.

**Conclusions.:**

Diagnostic evaluation of ARVC is challenging and requires multidisciplinary team collaboration. Further functional tests for elucidation of the clinical significance of the two novel variants of ARVC-associated genes could be suggested.

## Abstract (lt)

**Du nauji aritmogeninės dešiniojo skilvelio kardiomiopatijos genų variantai – atvejo aprašymas:**

**Santrauka. Apžvalga.**

Aritmogeninė dešiniojo skilvelio kardiomiopatija (ADSK) – paveldima širdies raumens liga, kuriai būdinga fibrozinė riebalinė dešiniojo skilvelio, kairiojo skilvelio ar abiejų skilvelių raumens degeneracija ir funkcijos sutrikimas. ADSK patogenezėje svarbi genetika, nes šią ligą sukelia genų, koduojančių desmosominius (*JUP*, *DSP*, *PKP2*, *DSG2*, *DSC2)* ir nedesmosominius baltymus, patogeniniai variantai, kurie yra viena iš dažniausių jaunų sportininkų staigios širdinės mirties (SŠM) priežasčių. Ankstyvas sergančių asmenų nustatymas, tinkamos prevencijos priemonės ir gydymas gali jiems išgelbėti gyvybę.

**Atvejo aprašymas.**

Šiame darbe pristatomas 65 metų vyro, kuriam buvo besikartojanti skilvelinė tachikardija (SkT), klinikinis atvejis. Pirmiausia buvo atmesta galima išeminė SkT priežastis. Atlikus ultragarsinį širdies tyrimą buvo nustatyti struktūriniai ir funkciniai dešiniojo skilvelio (DS) pakitimai. Įtarta ADSK. Diagnozei patikslinti buvo atliktas širdies magnetinio rezonanso tyrimas (MRT), kuriuo nustatyta sumažėjusi DS išstūmio frakcija su lokaliomis aneurizmomis, struktūriniai pakitimai dešiniojo ir kairiojo skilvelių miokarde. Atlikus genetinį ištyrimą patvirtinti nustatyti desmokolino-2 (*DSC2*) geno patogeninis variantas ir rianodino receptoriaus 2 (*RYR2*) geno neaiškios klinikinės reikšmės variantas.

**Išvados.**

ADSK diagnostika yra iššūkis gydytojui, reikėtų multidisciplininės komandos bendradarbiavimo. Reikia tolesnio ištyrimo, tai padėtų išsiaiškinti nustatytų genų variantų reikšmę.

**Raktažodžiai:** aritmogeninė dešiniojo skilvelio kardiomiopatija, skilvelinės aritmijos, desmosominių genų mutacijos.

## Introduction

Arrhythmogenic right ventricular cardiomyopathy (ARVC; ORPHA:247) is a chronic, progressive, heritable cardiomyopathy, characterized by fibrofatty replacement and dysfunction of right ventricular, left ventricular or both ventriculars muscle [1, 2]. It is one of the most common causes of sudden cardiac death (SCD) in young athletes, accounting for approximately 22 percent of cases [3]. Genetics plays an important role in pathogenesis of ARVC. It is caused by pathogenic variants of genes encoding desmosomal and non-desmosomal proteins [4]. The majority of the identified ARVC variants, classified as pathogenic or unknown in the disease genetic variant database, are in five most commonly mutated desmosomal protein genes: plakoglobin (*JUP*), desmoplakin (*DSP*), plakophilin-2 (*PKP2*), desmoglein (*DSG2*) and desmocollin-2 (*DSC2*) [5]. Desmosomal proteins dysfunction causes myocyte detachment from one another and cell death [6, 7]. It particularly happens when myocardium is placed under mechanical stress such as during physical activity [8, 9]. Also, there are number of non-desmosomal genes involved in ARVC-mimicking diseases: *TMEM43,* *CTNNA3*, *DES*, *LMNA*, *PLN, RYR2*, *TGFB3*, and *TTN *[1, 4, 10]. Early identification of patients, correct prevention and treatment can prevent SCD. The aim of this report is to present a 65-years-old man with an unusual clinical presentation of ARVC and discuss pathogenicity of the two novel identified variants in ARVC-associated genes.

## Case report

65-years-old man was admitted by ambulance to the Vilnius University Hospital Santaros klinikos after having palpitations for the first time in his life. The patients’ symptoms were accompanied by dizziness and weakness. Electrocardiogram (ECG) showed monomorphic ventricular tachycardia (VT) with left bundle-branch block morphology, heart rate was 202 beats per minute (BPM). Although Amiodarone infusion was administered to the patient, sinus rhythm was restored by synchronized electrical shock (150 J) due to the worsening of hemodynamic parameters. The man was monitored and observed in the hospital for the next 6 hours. No recurrent arrhythmia was observed and the next morning patient was discharged from the hospital.

**Fig 1.**
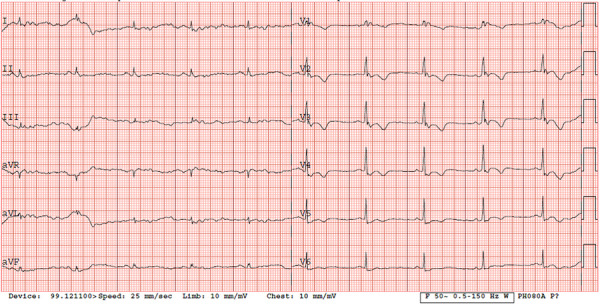
ECG during outpatient clinic cardiologist’s consulting: inverted T waves in precordial leads (V1, V2, V3, V4, V5) and the presence of complete right bundle-branch block – minor criteria according to the 2010 revised Task Force criteria (2). ECG – electrocardiogram.

Anamnesis revealed that the patient was consulted by a cardiologist in outpatient clinic three years ago due to episode of chest pain and dyspnea. ECG, lipidogram and exercise stress test were performed. ECG showed right bundle-branch block (RBBB) with T-wave inversion in V1-V5 leads (Fig. 1). According to the patient, ECG lesions have also been identified during previous preventive examinations. No pathological changes were observed in the lipidogram and exercise stress test. 

**Fig 2.**
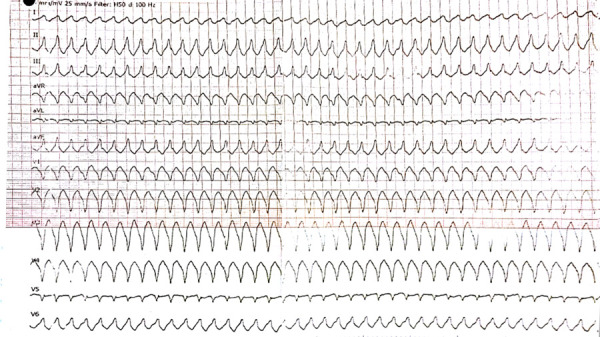
ECG performed in emergency room: VT with right ventricular (RV) outflow configuration, left bundle-branch block morphology with inferior axis (positive QRS in leads II, III, and aVF and negative in lead aVL) – minor criterion according 2010 revised Task Force criteria (2). ECG – electrocardiogram, RV – right ventricular, VT – ventricular tachycardia.

**Fig 3.**
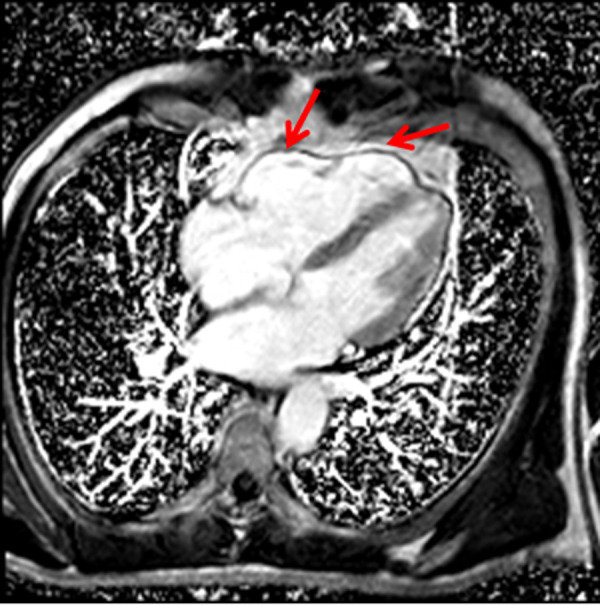
4 chamber LGE image. The RV local aneurysms has been reported in CMR. CMR – cardiac magnetic resonance, LGE – late gadolinium enhancement, RV – right ventricle.

**Fig 4.**
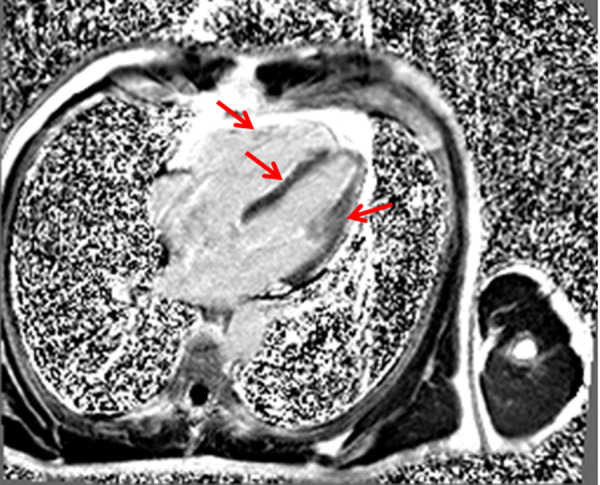
4 chamber LGE in the right and left ventricles. LGE – late gadolinium enhancement.

After 2 weeks the patient was admitted to the hospital again because palpitation and chest discomfort. ECG (Fig. 2), performed in emergency room, revealed the episode of monomorphic VT with left bundle-branch block morphology (heart rate 202 BPM). Sinus rhythm was restored by electrical cardioversion (70 J). Due to unclear cause of recurrent ventricular tachycardia the patient was hospitalized for detailed examination. The first thought was of acute coronary syndrome, therefore coronary angiography was performed showing no stenosis in coronary arteries. For the differential diagnosis of heart failure or cardiomyopathies, echocardiography was carried out. Right ventricular (RV) structural and functional abnormalities were found: trabeculation of RV, mild enlargement, decreased RV systolic function and relaxation, nevertheless left ventricular (LV) ejection fraction was normal (> 53%), no signs of LV dilatation. It was the first visualization method, which let us to suspect ARVC. Subsequently magnetic resonance imaging (MRI) was performed and its findings justified the suspicion of diagnosis of ARVC: segmental contraction with the local aneurysms has been reported, RV ejection fraction was 21% (figure 3). Additionally, structural changes ir the right (akinesia of RV inferior wall and diskinesia of RV apex) and left myocardium (regional akinesia of LV posterior wall and regional hypokinesia of LV lateral wall with preserved LV EF) has been reported (figure 4, 5 - late gadolinium enhancement (LGE) images – stuctural changes – LGE in the right and left ventricles). The MR radiologist conclusion was one of the major ARVC criteria (according to Task Force 2010). These findings suggested ARVC diagnosis of biventricular involvement.

Patient had one major (MRI) criterion and two minor (repolarization disorders ECG and ventricular arrhythmia) criteria of ARVC: complete right bundle-branch block in ECG with T-wave inversion in leads V1-V5 and VT with left bundle-branch block morphology. Genetic counseling assessment was performed for the patient. It was found out that the patient’s cousin died suddenly at the age of 40. According to the Task Force 2010 criteria, the diagnosis of ARVC was approved and it was decided to perform genetic test to confirm the molecular diagnosis of the disease.

**Fig 5.**
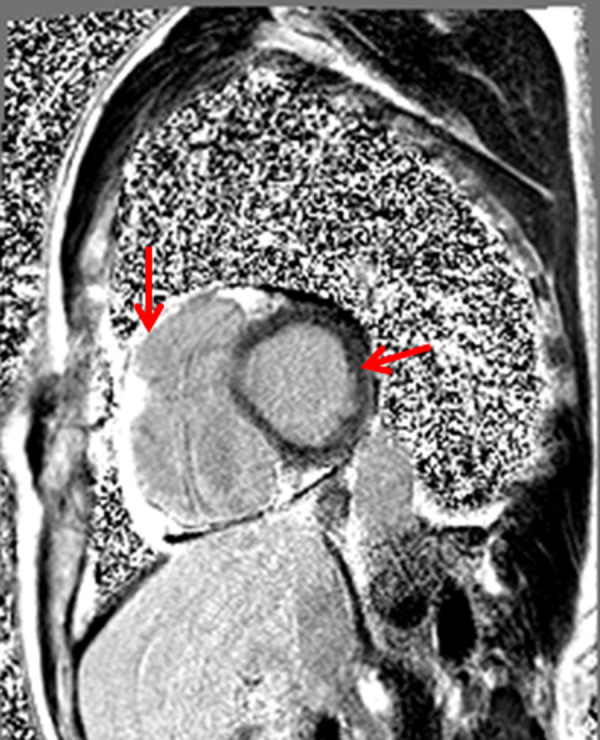
Short axis LGE in the right and left ventricles. LGE – late gadolinium enhancement

For SCD prevention and VT suppression extended-release metoprolol was prescribed and implantable cardioverter defibrillator (ICD) was implanted. 

After 8 months patient was admitted to emergency room due to VT (heart rate 180 BPM) without ICD discharge. Sinus rhythm was restored by electrical cardioversion (150J) and ICD was reprogrammed. The patient was subsequently treated with Amiodarone.

Next-generation sequencing analysis of genomic DNA isolated from patient’s peripheral blood was performed using TruSight Cardio Sequecing panel (Illumina Inc., San Diego, CA). Coding exons of 174 genes were analysed, including the main genes associated with ARVD (*DSC2, DSG2, DSP, JUP, PKP2,* *PKP2, RYR2, TGFB3, TMEM43*). A novel likely pathogenic *DSC2* gene (MIM#125645) variant NM_024422.3:c.577_624del, NP_077740.1:p. Gly193_Ser208del, rs767081975 and a variant of uncertain clinical significance in *RYR2* gene (MIM#180902) NM_001035.2:c.9018C>G, NP_001026.2:p. (Ser3006Arg) were identified. The other family members were not available for segregation analysis, since the patient has no children or siblings, his parents died because of cancer and heart failure at the age of 77 and 81. Only the fact of cousin sudden death at the age of 40, which could be associated with congenital heart disease, is known. 

Due to thyrotoxicosis, medical treatment with Amiodarone was changed to Metoprolol. Regular yearly follow-ups were planned for the patient.

## Discussion

Reasons of palpitations are various, but cardiac disorders are the most common cause of them. Cardiac etiology includes a wide spectrum of heart disorders: arrhythmias caused by acute and chronic ischemia, valvular heart disease, myocarditis, congenital heart diseases [11, 12], and – when the most common cardiac reasons are rejected, rare causes should be suspected – cardiomyopathies and channelopathies such as arrhythmogenic right ventricular cardiomyopathy (ARVC), congenital long QT syndrome (LQTS), catecholaminergic polymorphic ventricular tachycardia (CPVT), left ventricular noncompaction (LVNC), Brugada syndrome (BrS) [4]. All these conditions damage the heart muscle, the scar, fibrosis or fibro-fatty replacement are developed and these facts cause a regional slowing of action potentials, electrical reentry or abnormal automaticity [13]. Palpitations also can occur in the absence of a cardiac arrhythmia because of psychiatric (panic attacks, generalized anxiety disorder, somatization, depression) or miscellaneous causes (drug-induced, thyrotoxicosis, caffeine, cocaine, anemia) [11]. Nevertheless, these diagnoses should not be accepted until arrhythmic etiologies have been excluded. The basic screening of palpitations includes detailed history, physical examination, 12-lead ECG, laboratory testing and sometimes more extensive diagnostic testing for patients at high risk [14]: 24-hour Holter monitoring, exercise ECG testing, dobutamine stress echocardiography, cardiac MRI, coronarography or coronary CT angiography. Our patient was complaining of palpitations because of sustained ventricular tachycardia, which repeated several times in a short period. Acute coronary syndrome is the first diagnosis to be ruled out for ventricular arrhythmias. The next step is to reject myocarditis, heart failure and cardiomyophaties. Inverted T waves in precordial leads (V1-5) and the presence of complete right bundle-branch block in ECG suggested to look for right ventricular abnormalities. According to this fact, investigation of patient was extensive and involved imaging tests. The cause of ventricular arrhythmia was suspected when transthoracic echocardiogram and diagnosed when cardiac magnetic resonance was performed. 

The patient was older than the mean age at diagnosis for the most congenital heart disease (65-year-old), therefore this fact misled from suspecting genetic disorder during the first hospitalization. However, the characteristic features of ARVC allowed thinking about this disease later. Clinical manifestation of ARVC is the most common in approximately 30 years of age, however it varies between the age of 10 and 50 years [15–18]. Mean age at diagnosis of other congenital cardiomyopathies and channelopathies also varies, but typically they manifest until 40 years [4]. 

Several diagnostic tests are needed to evaluate diagnosis of ARVC. According to recommendations, basic screening includes family history, 12-lead ECG, transthoracic echocardiography and cardiac MRI in all patients with a suspected diagnosis of ARVC [14]. Echocardiographic criteria include evaluation of regional RV akinesia, dyskinesia or aneurysm, measures of RVOT enlargement and reduction in RV fractional area changed (FAC). MRI is recommended to all patients with suspected ARVC, because it plays the most important role in identification of global and regional ventricular dilatation or dysfunction, late gadolinium enhancement, focal wall thinning, intramyocardial fat. These two tools were the most important in detecting etiology of ARVC cardiac episode in the described case. 

ARVC diagnosis is based on diagnostic criteria, which were initially proposed by an international Task Force [McKenna et al 1994] [19], and were revised by Marcus et al [2010] [2]. Individuals are classified as having a definite, borderline, or possible diagnosis of ARVC based on the number of major and/or minor diagnostic criteria presented in 6 categories, including global or regional dysfunction and structural alterations, tissue characterization of wall, repolarization abnormalities, depolarization/conduction abnormalities, arrhythmias, and family history. Definite diagnosis of ARVC using the 2010 revised Task Force Criteria requires the presence of 2 major criteria or 1 major and 2 minor criteria or 4 minor criteria from different categories [2, 19]. Our patient has met 1 major and 2 minor criteria from three different categories for the definite diagnosis of ARVC [2, 19]. Additionally, identification of a likely pathogenic variant in *DSC2* gene gives additional major criterion and, in overall, our described patient has met 2 major and 2 minor criteria from four different categories. 

Genetic testing is important for familial cases and allows to estimate the risk for first-degree relatives, thus genetic counseling is strongly advised for family members of the proband with ARVC-causative mutation [4]. Data from literature suggest that 30 percent of cases of ARVC are familial [20–23]. *DSC2* gene alterations are identified in 2 to 7 percent of ARVC [4]. *DSC2* gene is found in a cluster with other desmocollin family members. Desmocollins, along with desmogleins, are cadherin-like transmembrane glycoproteins that are major components of the desmosomes which lead cells adhesion and are found in cells subject to mechanical stress. 

Despite the identification of the two novel variants in ARVC-associated genes, the assessment of their pathogenicity was not very straightforward. The variant c.577_624del, detected in our patient in *DSC2* gene has not been reported previously as pathogenic or as a benign polymorphism, to our knowledge. According to *in silico* analysis of pathogenicity, Mutation Taster algorithm predicted the identified**c.577_624del variant as disease causing (0,99). The variant results in the non-frameshift deletion of 16 amino acids in the Cadherin domain of the DSC2 protein p.(Gly193_Ser208del) [24]. Missense variants that are located within**the genomic region deleted in our patient (p.Asn194Lys, p.Arg203His, p. Arg203Cys) have been reported in the Human GeneMutation Database in association with ARVC [27-29], supporting the functional importance of this region of the protein. However, the ClinVar database classified (and does it so far) the variant as VUS making the evaluation of the variant slightly complicated. Still, according to ACMG criteria the inframe deletion scored at least 3 moderate and 1 supporting criteria of pathogenicity allowing us to attribute it to likely pathogenic class of variants. Nevertheless, a functional test would be required to confirm the pathogenicity of this alteration.

The evaluation of the contribution of the c.9018C>G variant in *RYR2* gene to the clinical features of our patient was also difficult. Cardiac ryanodine receptor (*RYR2*) gene mediates the release of diastolic calcium from the sarcoplasmic reticulum that is required for myocardial contraction.**Changes in this gene can trigger life-threatening ventricular arrhythmias and sudden cardiac death [25]. Mutations of *RYR2* are characteristic for patients with catecholaminergic polymorphic ventricular tachycardia – up to 60 percent of cases are related to this gene mutation [4]. The variant c.9018C>G detected in our patient was not described in the 1000 Genomes Project or ExAC database, what indicates it is not a common benign variant in these populations. However, this variant and any other variants in close positions have not been reported in the literature in individuals related with ARVC, and the predictions of pathogenicity using *in silico* tools are conflicting. Although the variant is extremely rare it is expected to change amino acid in functionally less active C terminus of the protein which does not belong to any domain of ryanodine receptor 2. Therefore, according to ACMG criteria we decided to classify the missense variant of *RYR2* gene as VUS (variant of unknown significance).

The main dangers of ARVC are the progression of the ventricular arrhythmias that cause SCD and the development of heart failure [26]. For these reasons, there are several key goals for the treatment of the disease [26]: reduction of mortality, limiting progression and symptoms of the disease, improvement of life quality. ARVC treatment also includes lifestyle changes, pharmacological treatment, catheter ablation, ICD implantation and heart transplantation, which are suggested for individuals with advanced end-stage heart failure or in case of uncontrolled ventricular tachycardia. The main tool for preventing SCD is the implantable cardioverter defibrillator (ICD). Our described patient was stratified as having high risk for major arrhythmic events [26], he had strong indication to implant ICD for SCD prevention [14, 26]. 

## Limitations

A major limit of this case report is the fact that there was no possibility to perform segregation analysis for the other family members of patient, because he has no children or siblings, his parents are dead. Extensive familial genetic testing could let to assess the pathogenicity of the two novel identified variants in ARVC-associated genes and to classify their clinical significance.

## Conclusion

Diagnostic evaluation of ARVC is challenging and requires multidisciplinary team collaboration. ECG, transthoracic echocardiogram and cardiac magnetic resonance imaging are very important for ventricular arrhythmias differential diagnosis. Subsequently performed genetic testing led to the identification of a novel ARVC likely pathogenic variant* in DSC2* gene and variant of uncertain significance in *RYR2* gene. Further functional tests for elucidation of the clinical significance of the variants could be suggested.
